# Leiomyoma cutis

**DOI:** 10.11604/pamj.2025.50.103.44518

**Published:** 2025-04-21

**Authors:** Aishwarya Kishor Kedar, Rohini Amar Rathod

**Affiliations:** 1 Department of Respiratory Medicine, Datta Meghe Institute of Higher Education and Research, Wardha, Maharashtra, India,; 2 Shri Bhausaheb Hire Government Medical College and Hospital, Dhule, Maharashtra, India

**Keywords:** Nodules, spindle cells, split skin thickness graft, cutaneous leiomyoma

## Image in medicine

A fifty-year-old male presented with complaints of multiple, painful, and localized swellings on the left arm for 5 years. To begin with, the swelling was single in number, elevated, erythematous, and associated with itching. It gradually increased in number and size over time and was associated with intense pain. There were no complaints of discharge or erosion at the site of swellings. There wasn’t any history of other cutaneous complaints as well. His systemic examination was within normal limits, and he had no comorbidities. No family history of similar complaints was present. Cutaneous examination revealed multiple, erythematous, tender, 1-2cm-sized, circular to oval-shaped, firm nodules over the unilateral left extensor surface of the arm. Nodules were not present elsewhere on the body. Nail and hair examination was within normal limits. Punch biopsy was taken from the site of the lesion and the histopathological report revealed the epidermis to be lined by stratified squamous epithelium, and the sub-epithelium had well-circumscribed tumour composed of interlacing bundles of spindle cells having cigar-shaped nuclei with tapered ends, small nucleoli, and indistinct cytoplasm. A diagnosis of cutaneous leiomyoma was made, and the patient was started on Tablet nifedipine 10mg once a day to relieve pain. As the pain was persistent even after 3 months, he was planned for excision and grafting. It was a split skin thickness graft taken from the thigh. After 3 months of graft implant, the patient had the formation of a keloid over the area, and the pain was relieved.

**Figure 1:**
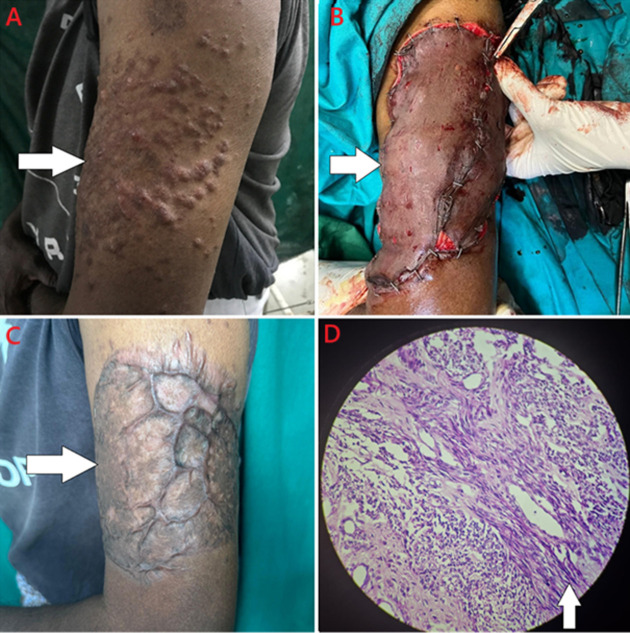
A) multiple, raised, erythematous, about 1-2cm-sized, circular to oval-shaped nodules over the unilateral left extensor surface of the arm; B) intra-operative image of split skin thickness graft; C) keloid formation over the area after 3 months; D) section studied showing interlacing bundles of spindle cells having cigar shaped nuclei with tapered ends, small nucleoli and indistinct cytoplasm

